# RIPK3 Induces Cardiomyocyte Necroptosis via Inhibition of AMPK-Parkin-Mitophagy in Cardiac Remodelling after Myocardial Infarction

**DOI:** 10.1155/2021/6635955

**Published:** 2021-03-27

**Authors:** Pingjun Zhu, Kun Wan, Ming Yin, Peng Hu, Yifan Que, Xin Zhou, Lei Zhang, Tianzhi Li, Yingzhen Du, Guogang Xu, Xiangqun Fang

**Affiliations:** ^1^Department of Respiratory and Critical Care Medicine, The Second Medical Center & National Clinical Research Center for Geriatric Diseases, Chinese PLA General Hospital, Beijing, China; ^2^Medical Supplies Center of Chinese PLA General Hospital, Beijing, China; ^3^Department of Emergency, The Second Medical Center & National Clinical Research Center for Geriatric Diseases, Chinese PLA General Hospital, Beijing, China; ^4^The Second Medical Center & National Clinical Research Center for Geriatric Diseases, Chinese PLA General Hospital, Beijing, China

## Abstract

Receptor-interacting protein 3- (RIPK3-) modulated necroptosis plays a critical role in cardiac remodelling after myocardial infarction (MI). However, the precise regulatory mechanism is not fully elucidated yet. In the present study, we showed that RIPK3 expression was upregulated in myocardial tissue after MI in a mouse model by coronary artery ligation, as well as in the cardiomyocytes following hypoxic injury *in vitro*. The increase of RIPK3 expression was found to be accompanied by severe cardiac remodelling, cardiac dysfunction, and higher mortality. Elevated RIPK3 expression subsequently abrogated the AMPK pathway that was accompanied by inhibition of Parkin-mediated mitophagy. Loss of mitophagy increased the opening of mitochondrial permeability transition pore (mPTP), which ultimately induced the cardiomyocyte necroptosis. In contrast, genetic ablation of *Ripk3* induced the AMPK/Parkin-mitophagy pathway, favouring a prosurvival state that eventually inhibited mPTP opening and induced the necroptosis of cardiomyocytes in the post-MI cardiac remodelling. In conclusion, our results revealed a key mechanism by which necroptosis could be mediated by RIPK3 via the AMPK/Parkin-mitophagy/mPTP opening axis, which provides a potential therapeutic target in the management of heart failure after MI.

## 1. Introduction

Today, ischemic heart failure remains a major health concern, accounting for high morbidity and mortality globally [1]. Myocardial infarction (MI) has been found to be the leading cause of ischemic heart failure. The loss of cardiomyocytes is confirmed as essential in the pathogenesis of cardiac remodelling following MI [2, 3], which is mainly due to necroptosis [4]. Thus, reducing cardiomyocyte necroptosis represents a very promising strategy in treating ischemic heart failure.

Necroptosis is one subtype of necrosis that is regulated by a specific pathway of cell death [5]. Specifically, the receptor-interacting protein 3- (RIPK3-) mediated mitochondrial permeability transition pore (mPTP) opening signalling pathways are key in regulating necroptosis of cardiomyocyte under different stimuli [4, 6, 7]. The mPTP opening causes proton gradient dissipation, which thereby modulates the oxidative phosphorylation and ATP synthesis, mitochondrial swelling and rupture, and eventually cardiomyocyte necroptosis [8, 9]. However, the precise mechanism by which RIPK3 regulates mPTP opening in post-MI cardiac remodelling remains elusive.

Mitochondria are intracellular organelles that are critical in certain intracellular processes that are fundamental in energy transformation with ATP [10]. Mitochondria are also important in cell life and fate as they pay key roles in initiating apoptosis. Maintaining homeostatic control of the mitochondria, in terms of both quantity and quality, is essential for cell fate and function [11]. Mitophagy is a protective mechanism which degrades those damaged mitochondria in the lysosomes under exposure to different stimuli [12–14]. Recent studies have confirmed that mitophagy is critically involved in the regulation of cellular necroptosis. In neuroblastic SH-SY5Y cells, activation of mitophagy by UNBS1450 inhibited the dissipation of mitochondrial membrane potential, mPTP opening, and cellular necroptosis. However, another study showed that mitophagy can also activate cellular necroptosis. During the progression in chronic obstructive pulmonary disease (COPD), inhibition of mitophagy was also shown to reduce the number of human pulmonary arterial endothelial cells undergoing necroptosis due to smoke exposure [15–17]. Thus, mitophagy appears to play different roles in the regulation of cell necroptosis in different contexts, which may be related to the cell type and pathological environment [18]. Accordingly, we were interested in determining the role of mitophagy in cardiomyocyte necroptosis during post-MI cardiac remodelling.

Numerous studies have shown that activation of mitophagy can inhibit mPTP opening [19–21]. The PINK1-dependent recruitment of Parkin to damaged mitochondria is crucial in the process of mitophagy [22, 23]. Parkin phosphorylation is necessary for the full activation of the protein's E3 ligase activity, which increases the capacity of Parkin to interact with LC3. At the molecular level, Parkin-mediated mitophagy inhibits mPTP opening by catalysing ubiquitination of CypD, a key component of the mPTP complex [24]. Furthermore, Parkin-dependent mitophagy was shown to be essential for cardiomyocyte repair in ischemia and reperfusion (I/R) injury [25], hypoxic injury [26], and cardiac hypertrophy [27], as well as diabetic cardiomyopathy [28].

Thus, we hypothesized that RIPK3 may cause Parkin-dependent mitophagy to provoke mPTP opening and cardiomyocyte necroptosis in the pathogenesis of cardiac remodelling after MI. To test this hypothesis, RIPK3-deficient mice (*Ripk3*^−/−^) were selected to establish an MI model with coronary artery ligation. We also established an *in vitro* model of hypoxic injury by using of cardiomyocytes from wild-type (WT) and *Ripk3*^−/−^ mice cultured in hypoxic conditions. The impact of RIPK3 after MI on mPTP opening and the expression of inflammatory factors were assessed. To explore the mechanisms underlying the influence of RIPK3 on post-MI heart remodelling, we evaluated the effects on angiogenesis, inflammation, oxidative stress, and cardiomyocyte necroptosis and the potential role of Parkin-mediated mitophagy on these effects.

## 2. Materials and Methods

### 2.1. Establishment of Animal Models

The present study was officially approved by the Institutional Animal Care and Use Committee, Chinese PLA General Hospital, Beijing. Gene knockout mice (*Ripk3*^−/−^) with a C57BL/6 background (male, 12 wk old, *n* = 60) were generated as previously described [19]. MI mouse models were established according to a standard protocol: prior to surgery, wild-type (WT, male, 12 wk old, *n* = 60) and *Ripk3*^−/−^ mice were administered with inhalation anaesthesia by using 1–2% isoflurane. The ligation surgical procedure of left anterior descending coronary artery was done as reported [29]. As a control, the specific artery remained intact in the sham group in the operation. Blood samples were collected 28 days after the surgery, emulating MI. The size of infarction was measured in the post-MI hearts using Masson's trichrome staining. Interstitial fibrosis in the noninfarct area was also investigated.

### 2.2. Determination of Cardiac Function

To monitor the cardiac function (*n* = 6/group) of animal models, echocardiography of all the mice was carefully examined by using a 14.0 MHz echocardiogram (Sequoia C512; Acuson) from Germany as reported in our previous study [19, 30].

### 2.3. Isolation of Cardiomyocytes and Primary Cell Culture, In Vitro I/R Injury Induction

Primary cardiomyocytes were all isolated from both *Ripk3*^−/−^ and wild-type (WT) mice according to our previous protocol [6]. The cells were subsequently proliferated in the high glucose-containing Dulbecco's modified Eagle medium (DMEM; Gibco) with 20% foetal bovine serum (FBS, HyClone, USA) in a 37°C incubation chamber supplied with 5% CO_2_ and 95% air. For the cardiomyocytes under *in vitro* I/R injury induction, cells were incubated in pure DMEM with no FBS and other ingredients in a hypoxia chamber (95% N_2_ and 5% CO_2_) for 24 h. The adenovirus plasmid (Ad-Parkin) and control adenovirus plasmid (Ad-ctrl) were purchased from Vigene Biosciences. The Ad-Parkin and Ad-ctrl were used to infect the cardiomyocytes.

### 2.4. Cellular Necroptosis Detection

Cell suspension was seeded in 6-well cell-culture plates at a density of 10^6^ cells/well and incubated at 37°C for 24 h. To identify cells that had undergone necroptosis, cardiomyocytes were stained with 5 *μ*L of FITC Annexin V and PI at RT (25°C) for 15 min in the dark with the FITC Annexin V Apoptosis Detection Kit (556547, BD Bioscience) [6].

### 2.5. Reactive Oxygen Species (ROS) Assays

ROS were measured after staining with dihydroethidium (DHE; Invitrogen, USA) [31]. The fluorescence was observed under microscopes and further analysed by flow cytometry. DHE was excited at 300 nm and 535 nm based on the standard protocol. The malondialdehyde (MDA) and the total superoxide dismutase (SOD) were detected as indicated by manufacturer (Biotein, Shanghai, China).

### 2.6. Enzyme-Linked Immunosorbent Assay (ELISA) of Cytokines

In addition to serum tumour necrosis factor-alpha (TNF-*α*) and interleukin-6 (IL-6), monocyte chemoattractant protein-1 (MCP-1) were also identified with commercially available ELISA kits from Technology Co., Ltd., Boster Bio, China.

### 2.7. RNA Isolation and Reverse Transcription-Quantitative PCR

RNA samples were extracted from the blood of mice or cells after treatment with hypoxia with routine laboratory protocol [32]. Total RNA (1 mg) was used for reverse transcription to synthesise cDNA (Invitrogen). Quantitative PCR was then run on a LightCycler 480 II system (Roche) with the LightCycler 480 Probes Master kit (Roche, 04887301001) and TaqMan primer-probe mix for each target gene. The reference gene 18S was used as an internal control.

### 2.8. Immunoblotting

For immunoblotting analysis, equal amounts (20–35 *μ*g) of total protein were fractionated by electrophoresis under denaturing conditions on a 4–20% sodium dodecyl sulphate- (SDS-) polyacrylamide gel (PAGE). The entire gel with protein bands was then transferred onto a piece of Immobilon-P polyvinylidene fluoride (PVDF) membrane (EMD Millipore, IPV00010). Since a gradient gel usually provides better separation of proteins with a wide range of molecular weights, we aimed to blot 2-3 proteins from the same membrane by cutting the membrane into small pieces. The target proteins were therefore ensured to fall into the middle of truncated membranes. In brief, the membranes were prestained with Pierce reversible protein stain kit and cut into 2-3 pieces based on the predicted molecular weights of target proteins according to direct visualization using a prestained protein ladder as reference. The PVDF membrane with proteins was blocked in 3% bovine serum albumin (BSA) for 1 h at RT. The membranes were further soaked overnight in primary antibodies at the desired dilution ratio in 3% BSA/TBST at 4°C. Primary anti-bodies for immunoblotting were as follows: PAGM5 (1 : 1000, Abcam, #ab126534), GADPH (1 : 1000, Abcam, #ab8245), p-MLKL (1 : 1000, Abcam, #ab196436), MLKL (1 : 1000, Cell Signaling Technology, #37705), Ripk3 (1 : 1000, Cell Signaling Technology, #95702), LC3II (1 : 1000, Cell Signaling Technology, #3868), p62 (1 : 1000, Abcam, #ab56416), Beclin1 (1 : 1000, Cell Signaling Technology, #3495), Atg5 (1 : 1000, Cell Signaling Technology, #12994), phospho-AMPK (Thr172, 1 : 500, Cell Signaling Technology, #2535), AMPK (1 : 1000, Cell Signaling Technology, #2532), Parkin (1 : 500, Abcam, #ab15954), phospho-Parkin (Ser65) (1 : 1000, Abcam, ab154995), and VDAC (1 : 1000, Abcam, #ab14734). They were subsequently washed in 1x TBST 5 min each for three times before incubating for 1 h with species-relevant horseradish peroxidase-conjugated secondary antibodies (1 : 5000–10,000) at RT. The PVDF membrane was washed again before being incubated in an Amersham ECL Prime Western Blotting Detection Reagent (GE Healthcare, RPN2232) or Clarity Max Western ECL Substrate (Bio-Rad, 1705062) as per the manufacturer's protocols, and target bands were detected on the ImageQuant LAS 400 system (GE Healthcare). The intensity of protein bands was quantified with the built-in ImageQuant TL software (Version 7.0 GE Healthcare) and normalized to the level of GADPH.

### 2.9. Immunofluorescence

Immunofluorescence analysis was performed on the cells as described previously. In brief, the cells were washed in phosphate-buffered saline (PBS) for three times prior to being fixed in 4% paraformaldehyde for 1 h at RT. Primary antibodies soaked with the cells were incubated overnight at 4°C. Afterwards, washout of excess primary antibodies (3 × 10 min with 0.1% Triton-X100 in PBS), the cells were further incubated for 2 h with secondary antibodies diluted in 5% NDS buffer at RT. The cover slips were subsequently washed with PBS-T (3 × 10 min) and PBS (1 × 10 min) in the dark and mounted onto slides using Fluoromount-G (SouthernBiothech, Birmingham, AL, USA). Immunostained preparations were analysed using a Nikon A1R confocal microscope (Melville, NY, USA) to determine protein expression.

### 2.10. Determination of mPTP Opening

mPTP opening was visualized by the rapid dissipation of tetramethylrhodamine ethyl ester (TMRE) fluorescence. We determined the arbitrary time of mPTP opening as the time when the TMRE fluorescence intensity was detected half-reduced between the initial and residual fluorescence intensities as reported. Cyclosporin A (CsA, 2 *μ*g/ml, Sigma Aldrich) was used as the inhibitor for mPTP opening in the negative control [33].

### 2.11. Statistical Analysis

One-way analysis of variance and the Student-Newman-Keuls test for post hoc comparisons were used to test for differences between the means of three or more independent groups. In addition, the Mann-Whitney *U* test was applied between the two experimental groups. The difference in the survival between two groups after MI was determined by the Mantel-Cox test. Data were all expressed as mean ± SEM. The statistical differences were cut off at *p* < 0.05.

## 3. Results

### 3.1. RIPK3 Aggravated Cardiac Remodelling after MI

**Figure 1:**
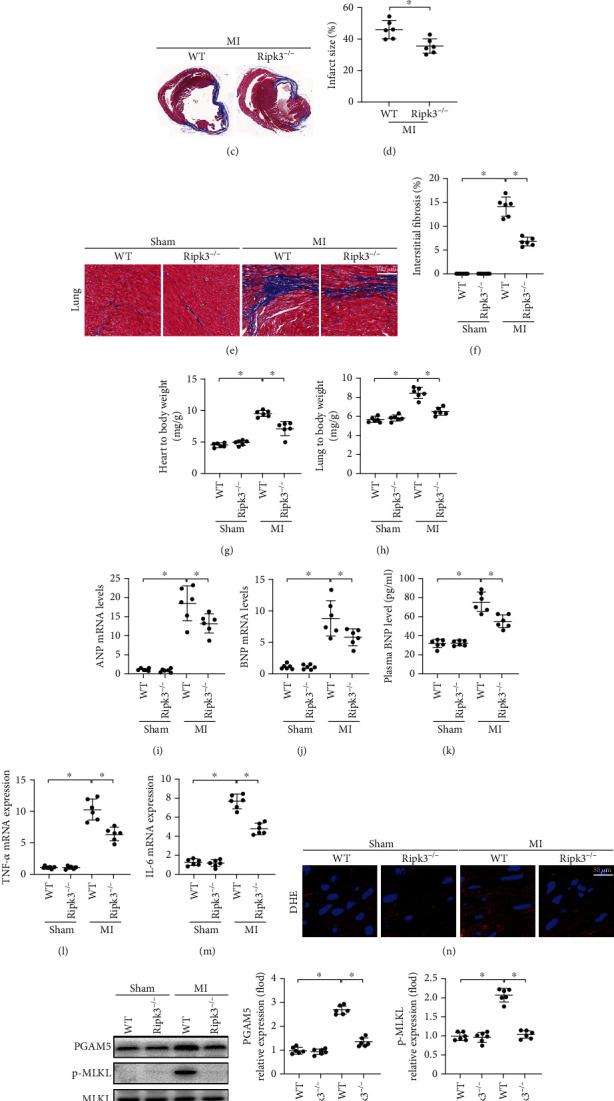
RIPK3 upregulation promoted cardiac remodelling after MI (*n* = 6/group). (a, b) The change of RIPK3 expression after MI. The infarct size (c, d) and the degree of fibrosis (e, f) of mice hearts were performed by Masson's trichrome staining. *Ripk3* genetic ablation reduced both the heart weight-to-body weight ratio (g) and the lung weight-to-body weight ratio (h). (i, j) The mRNA expression levels of myocardial atrial natriuretic peptide (ANP) and brain natriuretic peptide (BNP) were performed by RT-PCR. (k) The change of plasma BNP levels was detected by ELISA. (l, m) Loss of *Ripk3* reduced the mRNA levels of proinflammatory cytokines TNF-*α* and IL-6 in the peri-infarcted areas of the heart tissue. (n) ROS content in the heart tissue was detected by DHE staining. (o–q) The change of protein levels of phosphoglycerate mutase 5 (PGAM5) and phosphorylated mixed-lineage kinase domain-like protein (p-MLKL). Data are shown as the means ± SEM, ^∗^*p* < 0.05.

As shown in Figures 1(a) and 1(b), the western blot results demonstrated that expression of the myocardial RIPK3 protein was significantly upregulated in the peri-infarct heart tissue at 4 weeks after surgery compared to that of the sham-operated mice. Masson's trichrome staining of samples collected 4 weeks after MI showed that loss of *Ripk3* reduced the infarct size and the degree of fibrosis compared with those of the WT group after MI (Figures 1(c)–1(f)). Furthermore, *Ripk3* genetic ablation led to a lower level of heart weight-to-body weight ratio and lung weight-to-body weight ratio (Figures 1(g) and 1(h)). Gene knockout of *Ripk3* downregulated the mRNA expression in myocardial atrial natriuretic peptide (ANP), brain natriuretic peptide (BNP), and plasma BNP (Figures 1(i)–1(k)).

Compared with the WT group, deletion of the *Ripk3* gene induced lower mRNA expression levels of some proinflammatory cytokines such as TNF-*α* and IL-6 in the peri-infarcted areas of the heart tissue after MI (Figures 1(l) and 1(m)). DHE staining showed that the ROS content was elevated in the WT group but was restored to normal levels in the *Ripk3*^−/−^ group after MI (Figure 1(n)). In addition, decreased SOD enzymatic activity and increased content of MDA in remodelled hearts was reversed by *Ripk3* deletion (Supplemental Fig. 1A–B). *Ripk3* genetic deletion was also found to show effects of attenuating cardiac necroptosis, as confirmed by the decreased levels of phosphoglycerate mutase 5 (PGAM5) and phosphorylated mixed-lineage kinase domain-like protein (p-MLKL) (Figures 1(o)–1(q)). Collectively, these results indicated that RIPK3 contributed to post-MI heart remodelling.

### 3.2. Ablation of Ripk3 Gene Enhanced the Cardiac Function and Survival of MI Mice

**Figure 2:**
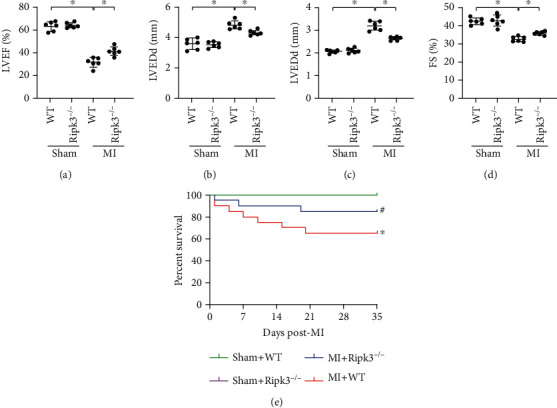
Genetic ablation of *Ripk3* enhanced the cardiac function and survival of MI mice. (a–d) Cardiac function is evaluated through echocardiography (*n* = 6/group). Data are shown as the means ± SEM, ^∗^*p* < 0.05. LVEF: left-ventricular ejection fraction; LVFS: left-ventricular fractional shortening; LVEDd: left ventricular end-diastolic diameter; LVESd: left ventricular end-systolic diameter. (e) Survival of WT or *Ripk3^−/−^* mice treated with sham or MI surgery (*n* = 20/group), ^∗^*p* < 0.05 vs. Sham+WT group, ^#^*p* < 0.05 vs. MI+WT group.

Echocardiography analysis at 4 weeks after MI confirmed that knockout of *Ripk3* significantly improved the left ventricular ejection fraction (LVEF) and left ventricular fractional shortening (LVFS) and also reduced the internal diameter of the left ventricle at the end of systole and diastole (Figures 2(a)–2(d)). In sham-operated mice, the ablation of *Ripk3* had no effects on the above parameters in echocardiography scans. Furthermore, *Ripk3^−/−^* mice had higher survival rates after MI compared with that of WT mice (Figure 2(e)). Taken together, our results strongly indicate that knockout of the *Ripk3* gene would help maintain the cardiac performance and survival during the chronic phase of MI.

### 3.3. RIPK3-Induced Necroptosis Is Mediated by mPTP Opening in Cardiomyocytes

**Figure 3:**
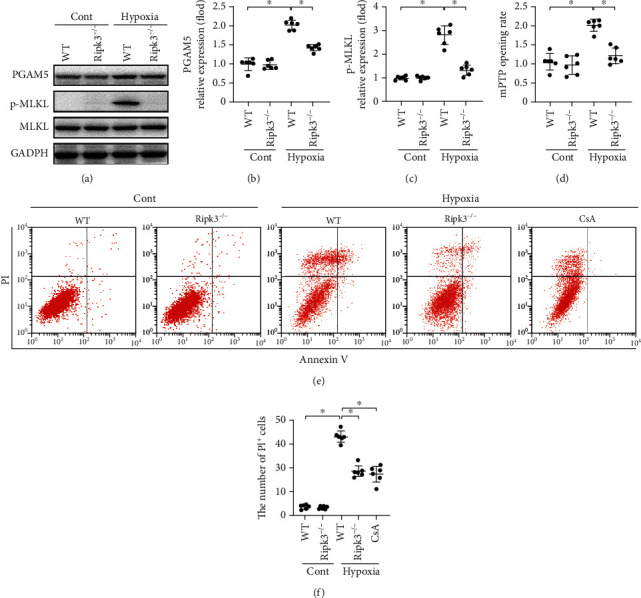
RIPK3 initiated necroptosis via promoting mPTP opening (*n* = 6/group). (a–c) The expression change of PGAM5 and p-MLKL was determined by western blot in vitro. (d) The change of mPTP opening time. (e) The cardiomyocyte necroptosis was measured flow cytometry with Annexin V/PI staining. Necroptosis group: the percentage of PI^+^ cells. Data are shown as the means ± SEM, ^∗^*p* < 0.05.

As shown in Figures 3(a) and 3(b), the cardiomyocyte necroptosis index was increased by hypoxia treatment for 24 h, based on the elevated expression of PGAM5 and p-MLKL. However, this effect was reversed in cardiomyocytes from *Ripk3^−/−^*mice. To probe the underlying mechanism of *Ripk3*-mediated cardiomyocyte necroptosis, we first evaluated the rate of mitochondrial mPTP opening. Hypoxia treatment increased the mPTP opening time in WT cells, which was reversed in the case of *Ripk3* deficiency (Figure 3(d)). Inhibition of mPTP opening with CsA reduced necroptosis of WT cells under hypoxic stress, reaching a similar level to that as observed under *Ripk3* deletion (Figures 3(e) and 3(f)). Thus, mPTP opening plays a role in *Ripk3*-induced cardiomyocyte necroptosis.

### 3.4. RIPK3 Promoted mPTP Opening via Reducing Parkin-Related Mitophagy

**Figure 4:**
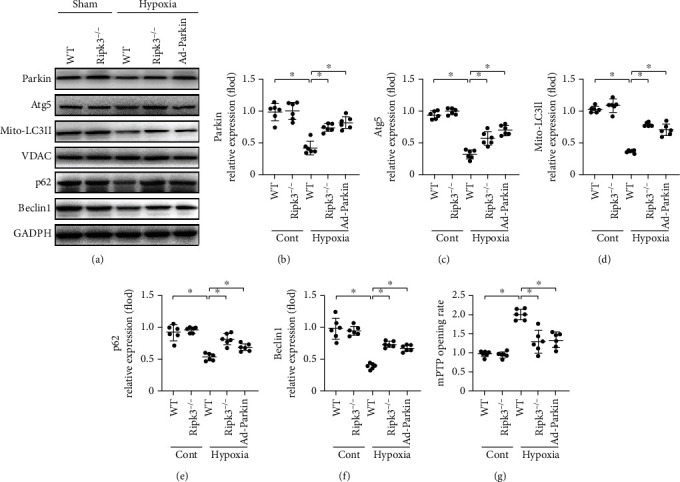
RIPK3 induced mPTP opening via Parkin-related mitophagy (*n* = 6/group). (a–f) Western blots were used to analyse the protein related to mitophagy. The gain-of-function assay about Parkin was carried out via adenovirus vector (Ad-Parkin) transfection. (g) The change of mPTP opening time. Data are shown as the means ± SEM, ^∗^*p* < 0.05.

As shown in Figures 4(a)–4(f), hypoxia reduced the levels of mitophagy markers in WT cells, including mito-LC3II, Atg5, Beclin1, and p62, compared with those of the control group cultured under normoxic conditions. However, these changes were reversed by *Ripk3* deletion. Since Parkin is essential for cardiac repair in post-MI heart remodelling, we further focused on the role of Parkin-related mitophagy in the *Ripk3*-mediated regulation of mitophagy under hypoxic stress. A gain-of-function Parkin assay was performed by overexpressing Parkin in the cardiomyocytes via transfection with an adenovirus vector (Ad-Parkin). Overexpression of Parkin reduced the hypoxic-induced mPTP opening following hypoxia injury in WT cells, which was consistent with the effect of *Ripk3* ablation (Figure 4(g)). These data confirmed that RIPK3 regulates mPTP opening via Parkin-related mitophagy.

### 3.5. RIPK3 Regulated Parkin-Related Mitophagy via the AMPK Pathway

**Figure 5:**
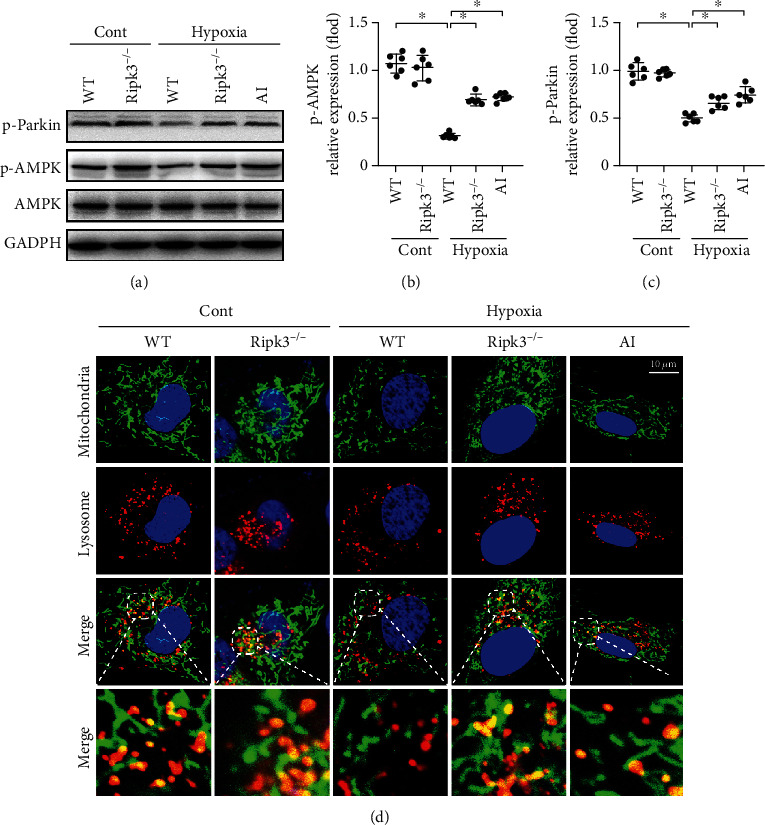
RIPK3 inactivated Parkin via inhibiting AMPK-mediated Parkin phosphorylation (*n* = 6/group). (a–c) Parkin and AMPK phosphorylation was analysed by western blots. AICAR (AI), the AMPK pathway activator, was used to activate AMPK pathways in WT cell under hypoxia injury. (d) The coimmunofluorescence of mitochondria and lysosomes in cardiomyocyte. AMPK activation facilitated the disassociation between the mitochondria and lysosome under hypoxia injury. Data are shown as the means ± SEM, ^∗^*p* < 0.05.

Hypoxic injury reduced the level of Parkin phosphorylation. However, this change was prevented with *Ripk3* deletion. To further investigate AMPK regulation of Parkin phosphorylation, we also observed the levels of AMPK activation under hypoxia. As shown in Figure 5(a), AMPK phosphorylation was suppressed under hypoxic conditions; this was reversed by *Ripk3* knockout. Treatment of the cardiomyocytes with the AMPK pathway activator (AICAR, AI) facilitated the phosphorylation of AMPK in the hypoxia group, together with increased Parkin phosphorylation (Figures 5(a)–5(c)). Costaining of the mitochondria and lysosome revealed that AMPK activation declined the association between the mitochondria and lysosome under hypoxic injury (Figure 5(d)). These data confirmed our hypothesis that RIPK3 regulates Parkin-related mitophagy via the AMPK pathway.

## 4. Discussion

Loss of cardiomyocytes after myocardial infarction promotes cardiac remodelling. RIPK3 supposedly plays a key role in cardiomyocyte necroptosis after MI. In our study, it was found that (i) RIPK3 could be activated after MI and would facilitate the development of post-MI cardiac remodelling; (ii) genetic ablation of *Ripk3* alleviated cardiac remodelling and promoted the cardiac function and survival of MI mice; (iii) *Ripk3* upregulation in cardiomyocytes inhibited AMPK phosphorylation, which reduced Parkin activation, thereby inhibiting mitophagy; and (iv) inactivation of mitophagy promoted opening of the mPTP, leading to cardiomyocyte necroptosis.

Mechanistically, formation of the “necrosome” by RIPK1, RIPK3, and p-MLKL results in necroptosis in various types of cells. However, myocardial necroptosis was reported to be the prominent mode of cell death, which is dependent on RIPK3 and mPTP opening [4, 6, 19]. Although the potential mechanism of how the mPTP opening triggers cardiomyocyte necroptosis is well understood due to extensive research, the precise mechanism by which RIPK3 triggers mPTP opening has remained elusive, especially during cardiac remodelling after MI. Therefore, this study fills this gap in knowledge by confirming that RIPK3 induces necroptosis via the AMPK/Parkin-mitophagy/mPTP opening pathway, representing a novel signalling pathway involving post-MI cardiac remodelling as well as cardiomyocyte necroptosis.

The current study identified that mitophagy could act as a negative regulator of cardiomyocyte necroptosis in post-MI cardiac remodelling. Although accumulating evidence has hinted at a potential association between mitophagy and necroptosis, the regulatory role of mitophagy in necroptosis activation remains controversial. In patients with COPD, activation of both mitophagy and necroptosis has been observed in lung epithelial cells under persistent cigarette smoke extract (CSE) stimulation [34]. However, in Crohn's disease, activation of mitophagy was found to inhibit RIPK3-dependent necroptosis in the intestinal epithelium [35]. Therefore, the cross-talk between mitophagy and necroptosis might differ depending on the stimulus exposure and disease model.

Extensive research on Parkin-dependent mitophagy has been carried out in the field of cardiac injury [24, 36]. Our results demonstrated that an increase in Parkin-related mitophagy was accompanied by a decrease in mPTP opening and cardiomyocyte death, which was consistent with previous studies. In addition, mitophagy could be activated by specific receptors such as Fundc1, Bnip3, and Parkin [37, 38]. The role of other mitophagy-related receptors in regulating cardiomyocyte necroptosis should be taken into consideration for further research.

In addition, AMPK plays a protective role in a variety of pathophysiological processes in cardiac functions [39]. AMPK activates Parkin-mediated mitophagy through the phosphorylation of PINK1 and thus modulates Parkin recruitment/activation from the cytoplasm to the outer membrane of mitochondria [18, 40, 41]. In the current study, we identified RIPK3 as a negative regulator of Parkin-mediated mitophagy via inhibiting the AMPK pathway in cardiomyocytes, thereby revealing a novel myocardial necroptosis regulation model. However, the regulation mechanism of AMPK by RIPK3 warrants further investigation.

## 5. Conclusion

In conclusion, hypoxic injury and MI were associated with increased RIPK3 expression, leading to inactivation of AMPK. The reduction in AMPK in turn inhibited Parkin activation, which suppressed mitophagy and promoted mPTP opening and cellular necroptosis after MI. Thus, genetic ablation of *Ripk3* could activate the AMPK/Parkin-mitophagy pathway and subsequently prevent mPTP opening and cardiomyocyte necroptosis, ultimately preventing cardiac remodelling. Our results provide a potential therapeutic target to treat ischemic heart failure after MI.

## Data Availability

The original data presented in the study are included in the article; further inquiries can be directed to the corresponding author/s.

## Supplementary Materials

Supplementary Materials Supplemental Figure: total SOD activity (A) and MDA level (B) in hearts (*n* = 6/group); data are shown as the means ± SEM, ^∗^*p* < 0.05.
