# Supplementary figures and images for: RIPK3 Induces Cardiomyocyte Necroptosis via Inhibition of AMPK-Parkin-Mitophagy in Cardiac Remodelling after Myocardial Infarction
Source: Oxid Med Cell Longev. 2021 Mar 27;2021:6635955. doi: 10.1155/2021/6635955 (PMC8019651; doi:10.1155/2021/6635955)

# Supplemental Figure

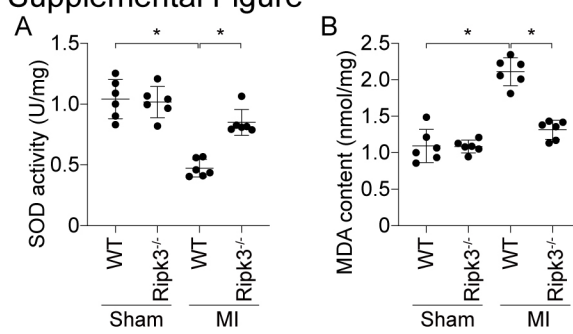

Supplement: Supplementary Materials — Supplemental Figure: total SOD activity (A) and MDA level (B) in hearts (n = 6/group); data are shown as the means ± SEM, ∗p < 0.05. [file 6635955.f1.pdf]
